# Pharmacokinetics of Intravenous and Oral Phenobarbital Sodium in Healthy Goats

**DOI:** 10.3389/fvets.2020.00086

**Published:** 2020-02-21

**Authors:** Liana M. Yates, Monica Aleman, Heather K. Knych, Marguerite F. Knipe, Chelsea M. Crowe, Munashe Chigerwe

**Affiliations:** ^1^William R. Pritchard Veterinary Medical Teaching Hospital, School of Veterinary Medicine, University of California, Davis, Davis, CA, United States; ^2^Department of Veterinary Medicine and Epidemiology, School of Veterinary Medicine, University of California, Davis, Davis, CA, United States; ^3^K. L. Maddy Equine Analytical Chemistry Laboratory, School of Veterinary Medicine, University of California, Davis, Davis, CA, United States; ^4^Department of Surgical and Radiological Sciences, School of Veterinary Medicine, University of California, Davis, Davis, CA, United States

**Keywords:** goat, phenobarbital, pharmacokinetics, brain, sleep, vigilance, electroencephalogram

## Abstract

Phenobarbital is a common drug used to manage epilepsy in goats. However, the recommended dose and dosing frequency are based on studies in dogs and horses. Studies describing the pharmacokinetics of phenobarbital when administered orally and assessing changes in behavior with concurrent electroencephalogram (EEG) readings are warranted in goats. The objectives of this study were to determine the bioavailability of orally administered phenobarbital and determine the effect of phenobarbital on brain activity using EEG in healthy goats. A cross-over design with 8 non-pregnant goats was performed. The goats were administered phenobarbital intravenously at 10 mg/kg, followed by a 2 week wash out period, and then administered phenobarbital, orally, at 10 mg/kg. Plasma sample determination of phenobarbital concentrations were collected at 13 time points. Continuous EEG readings with simultaneous video recording for 12 h was performed to determine the state of vigilance using a behavior scoring system prior to and after phenobarbital administration. Bioavailability of phenobarbital was 24.9%. Mean ± SD for half-life was similar between the oral (3.80 ± 0.826 h) and intravenous (4.0 ± 0.619 h) routes. Time to observed maximum concentration (T_max_), and maximum plasma concentration (C_max_) for the oral administration were 1.75 ± 0.46 h and 4,478.7 ± 962.4 ng/mL, respectively. Clearance was 152.5 ± 102.7 ml/h/kg. Area under the curve from zero to infinity (AUC_0→∞_) was 155,813 ± 218,448 and 38,763 ± 9,832 h^*^ng/mL for the intravenous and oral administration routes, respectively. Behavior score at 3 h after phenobarbital administration was different (*P* = 0.0002) from the score prior to administration for the oral administration route. In contrast, behavior scores before administration of phenobarbital and each time point after administration were not different (*P* >0.05) for the intravenous administration route or other oral administration route time points. Bioavailability of phenobarbital was poor, and the half-life was very short due to a high clearance. Doses >10 mg/kg should be considered when phenobarbital is administered orally in goats.

## Introduction

Phenobarbital is a common anti-epileptic drug administered in veterinary species for its primary potentiation of the action of GABA on the GABA_A_ receptor resulting in neuronal hyperpolarization (1). Although the pharmacokinetics of phenobarbital administered orally have been investigated in dogs (2), horses (3), and some species of birds (4), no peer reviewed studies are available in ruminants. In clinical practice, phenobarbital is administered orally to manage epilepsy in goats (5). Veterinarians administer phenobarbital orally in goats to manage epilepsy at various doses and dosing frequency intervals based on recommendations for monogastric species such as horses and dogs, or response to treatment. The rumen microbes in ruminants may degrade the orally administered phenobarbital; therefore, dose and dosing frequencies recommended for monogastric species cannot be easily extrapolated to ruminants. Thus, doses recommended in monogastric species might be insufficient or unsafe for ruminants. Oral administration of phenobarbital is practical and financially feasible for veterinarians and clients managing goats with epilepsy. Therefore, studies on bioavailability of orally administered phenobarbital are warranted.

Electroencephalogram (EEG) is a well-established diagnostic tool to assess cerebral cortical function. In goats, EEG has been used to monitor change in behavior following administration of barbiturates (6), assess rumination (7), and monitor anesthetic depth (8). Administration of phenobarbital can affect brain activity and behavior in goats. However, there is a dearth of information focusing on monitoring brain activity using EEG following phenobarbital administration. Studies describing the pharmacokinetics of phenobarbital in healthy goats are required to establish baseline information on dose, dosing frequency, and monitoring of brain activity to guide further studies in the management of goats with epilepsy. We hypothesized the following: **(**1) oral administration of phenobarbital in goats reaches similar total exposure (area under the curve) comparable to those achieved by the intravenous route and; **(**2) administration of phenobarbital orally or intravenously causes a change in brain electrical activity as determined by EEG. The objectives of this study were to determine the bioavailability of orally administered phenobarbital and determine the effect of phenobarbital on the EEG.

## Materials and Methods

### Animals and Experimental Design

Sample size calculation was performed using a commercial statistical software (JMP Pro v14, SAS Institute, Cary, NC, USA). Sample size calculation was based on testing differences among means of phenobarbital concentrations at different time points after oral administration. Using the mean and standard deviations at different time points after oral dosing of phenobarbital reported in horses (3, 9), a type 1 error of 0.05, and a power of 80%, it was determined that a minimum of 8 goats was required. Eight, non-pregnant, 1-year-old female goats (6 Saanens and 2 Alpines) from the University of California Davis (UCD) Dairy Goat Barn herd were randomly selected for a cross-over study design, with a 2-week wash-out period. The goats were transported to the University of California Davis Livestock Medicine and Surgery service of the teaching hospital and housed in a single goat pen. Four goats were enrolled at a time. The goats were allowed to acclimatize to the hospital environment for 2 days. The goats were deemed clinically and neurologically healthy based on clinical examination. The goats were weighed, and blood was collected for serum biochemical analysis to assess liver function prior to procedures. The study was performed from June to August 2019.

### Intravenous Administration of Phenobarbital

Prior to procedures, a 16-gauge × 3.25-inch intravenous (IV) catheter (Mila catheter extended use, Mila International Inc., Florence, KY, USA) was placed in the left or right external jugular vein, after sterile preparation of the catheter site. The IV catheter was secured by a non-absorbable suture material (Supramid size 0, S. Jackson Inc., Alexandria, VA, USA) and a bandage wrap. Phenobarbital sodium (Phenobarbital sodium injection 130 mg/mL, West-Ward Pharmaceutical, Eatontown, NJ, USA) was administered at 10 mg/kg, once, through the IV catheter. The catheter was then flushed with 4–6 ml of heparinized saline to ensure complete drug infusion. The dose of 10 mg/kg was chosen based on anecdotal recommendations for goats (10). Blood samples (6 mL) in tubes containing lithium heparin as an anticoagulant (Becton-Dickinson lithium-heparin tubes, Franklin Lakes, NJ, USA) were collected from the intravenous catheter at 0 (prior to administration of phenobarbital sodium), 0.25, 0.5, 0.75, 1.0, 2, 4, 6, 8, 12, 16, 20, and 24 h. To prevent contamination of the blood samples at each collection time point, 6 mL of blood was scavenged prior to collection of samples intended for analysis. Heparinized saline (4–6 mL) was used to flush the IV catheters when necessary to maintain patency. Daily clinical examination procedures including rectal temperature assessment, heart rate, respiratory rate, appetite, and demeanor were performed. The IV catheter was removed after the sample collection at the 24 h time point. Blood samples were then stored at 4°C and plasma was harvested after centrifugation at 2,800 × *g* within 12 h after collection. Plasma samples were then stored at −20°C until phenobarbital concentration determination. The goats were transported back and housed at the UCD Dairy Goat Barn during the 2-week washout period.

### Oral Administration of Phenobarbital

Following the 2-week washout period, the goats were transported back to the UCD Livestock Medicine and Surgery service. Procedures prior to the oral (PO) administration of phenobarbital were similar to the IV administration route procedures. Phenobarbital tablets at 10 mg/kg (Phenobarbital tablets 60 mg, West-Ward Pharmaceutical, Eatontown, NJ, USA) were administered orally. The tablets were crushed and mixed thoroughly with 60 mL of water, and then administered with a dosing syringe to ensure complete administration of the drug. Procedures after oral administration of phenobarbital were similar to the IV route procedures.

### Electrophysiologic Study

**Figure 1:**
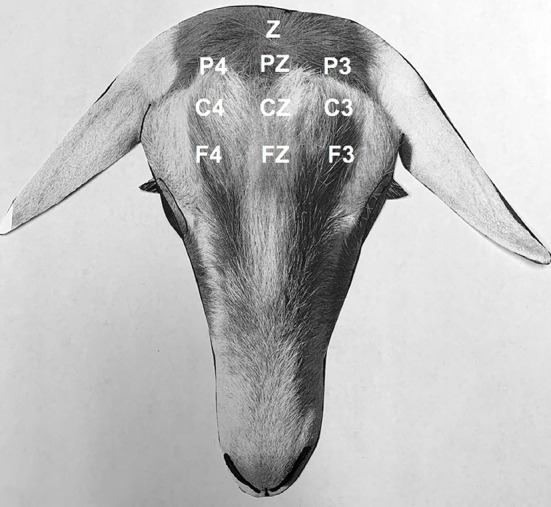
Electroencephalogram. Bipolar montage (transverse, rostral to caudal) and electrode placement in goats: F4, right frontal; Fz, frontal vertex; F3, left frontal; C4, right central; Cz, central vertex; C3, left central; P4, right parietal; Pz, parietal vertex; P3, left parietal; Z, ground. Odd numbers designate the left side, even numbers designate the right side, and z designate the midline.

After placement of the IV catheter for intravenous or oral phenobarbital administration procedures, the hair around the pole of the head was clipped, and 25-gauge subdermal wire electrodes (Ives EEG Solutions, Newburyport, MA, USA) were placed subcutaneously in the scalp with the goat standing, without sedation, for EEG recording. A transverse and rostral to caudal bipolar montage was used with the following channels; F3-Fz, Fz-F4, C3-Cz, Cz-C4, P3-Pz, Pz-P4, F3-C3, C3-P3, Fz-Cz, Cz-Pz, F4-C4, C4-P4, and Z; where F = frontal, C = central, P = parietal, and Z = ground electrode (Figure 1). The needle electrodes were secured using tape and adherent povidone-iodine dressing. A vest with a pouch was placed around the goats' thorax, and the EEG machine was placed in the vest pouch and secured around the goats' thorax with bandaging material. A Cadwell digital ambulatory wireless EEG system (Cadwell ArcEEG, Kennewick, WA, USA) with integrated video was used to obtain all standard EEG recordings. A baseline EEG recording was performed prior to the administration of phenobarbital. To maintain visual social interaction between the goat under study and the other three goats, a non-solid panel fencing was used to separate the animals.

Continuous EEG with simultaneous video recording for 12 h were performed to determine state of vigilance at various time points. Each time point of evaluation included six, consecutive, 10 s epochs for a total of 60 s. Time points were defined as; BL = baseline (prior to phenobarbital administration), PB = phenobarbital administration, T15 = 15 min post administration, T30 = 30 min, T45 = 45 min, T1 = 1 h, T2 = 2 h, T3 = 3 h, T4 = 4 h, T5 = 5 h, T6 = 6 h, T7 = 7 h, T8 = 8 h, T9 = 9 h, T10 = 10 h, T11 = 11 h, and T12 = 12 h. States of vigilance were defined by visual examination of the raw EEG recording concurrently with observation of the goat's behavior. Frequency bands were defined as; gamma (γ, >30 Hz), beta (β, 13 to <30 Hz), alpha (α, 8 to <13 Hz), theta (θ, 4 to <8 Hz), and delta (δ, >0 to <4 Hz). For data analysis purposes, the states of vigilance were defined and scored as follows; Score 1 = Awake moving (EEG; high frequency, low amplitude, mixed with gamma frequency, or non-interpretable EEG due to movement or chewing), 2 = Quiet sternal (high frequency, low amplitude with no gamma frequency), 3 = Drowsy (quiet sternal with eyes semi-closed, high frequency, low amplitude with some slowing of the waves), 4 = Transients of sleep (defined as isolated waves distinguished from background activity, and predominant slowing of the waves), 5 = Slow wave sleep (SWS; slow waves, sleep spindles, delta frequency, recumbency), 6 = Rapid eye movement sleep (REM; high frequency, low amplitude with rhythmic rapid eye movements, recumbency with head supported on ground, fence or other pen structures, and lack of muscle tone).

### Pharmacokinetic Analysis

The concentration of phenobarbital was measured in plasma by liquid chromatography tandem-mass spectrometry (LC-MS/MS) using negative electrospray ionization. Briefly, phenobarbital working solutions were prepared by dilution of the 1 mg/mL stock solution (d5 phenobarbital, Cerilliant, Round Rock, Texas, USA) with methanol to concentrations of 0.01, 0.1, 1, and 10 ng/μL. Plasma calibrators were prepared by dilution of the working standard solutions with drug free plasma to concentrations ranging from 0.2 to 30,000 ng/mL. Calibration curves and negative control samples were prepared fresh for each quantitative assay. In addition, quality control samples (plasma fortified with analyte at three concentrations within the standard curve) were included with each sample set as an additional check of accuracy. The response for phenobarbital was linear and produced correlation coefficients (*R*^2^) of ≥ 0.99. The precision and accuracy of the assay were determined by assaying quality control samples in replicates (*n* = 6) for phenobarbital. Accuracy was reported as percent nominal concentration and precision was reported as percent relative standard deviation. Accuracy for phenobarbital determination was 102% for 0.6 ng/mL, 98% for 40 ng/mL, and 95% for 400 ng/mL. Precision was 5% for 0.6 ng/mL, 2% for 40 ng/mL, and 4% for 400 ng/mL. The technique was optimized to provide a limit of quantitation of 0.2 ng/mL and a limit of detection of approximately 0.05 ng/mL for phenobarbital.

### Statistical Analysis

Pharmacokinetic parameters were determined using a non-compartmental model using a commercial software (Phoenix WinNonlin v8.1, Certara, Princeton, New Jersey, USA). The phenobarbital concentrations were plotted against time for both routes of administration. The maximum (peak) concentration (C_max_) and time to reach maximum concentration (T_max_) for the PO administration route were based on visual inspection of the concentration-time data. The slope of the terminal portion of the curve (log transformed concentrations), lambda *z* (λ_*z*_) was used to calculate half-life (HL λ_z_) using the equation 0.693/λ_*z*_ for both routes of administration. The area under curve (AUC) from time 0 to infinity (AUC_0_ → ∞) for both routes of administration were determined using the log-linear trapezoidal rule. Clearance (Cl) and the apparent volume of distribution at steady state (V_ss_) were determined by the pharmacokinetic software using the following formulas:

$$\begin{array}{l} {Cl=Dose/AUC}_{0}\to \infty \\ V_{ss}=MRT_{inf}xCl \end{array}$$

*Where MRT is the mean residence time*.

Bioavailability (F) was calculated using the formula:

$$\begin{array}{l} F=AUC_{oral}\;÷\;AUC_{iv}. \end{array}$$

Behavior of the goats based on video recording and concurrent EEG recordings at each time point were summarized as median (range) scores. For each route of phenobarbital administration, the outcome of interest was the association between the behavior scores prior to administration compared to behavior scores at each time point after phenobarbital administration. The association between the behavior scores before phenobarbital administration (BL) and each time point after phenobarbital administration was determined by a non-parametric one-way analysis of variance using the Friedman test and *post-hoc* analysis using the Dunn's test, with *p*-value adjustment for multiple comparisons. Commercial statistical softwares (GraphPad Prism v8, San Diego, CA, USA; JMP Pro v14, SAS Institute, Cary, NC, USA) were used to analyze the data. For all analyses *P* < 0.05 was considered significant.

## Results

**Figure 2:**
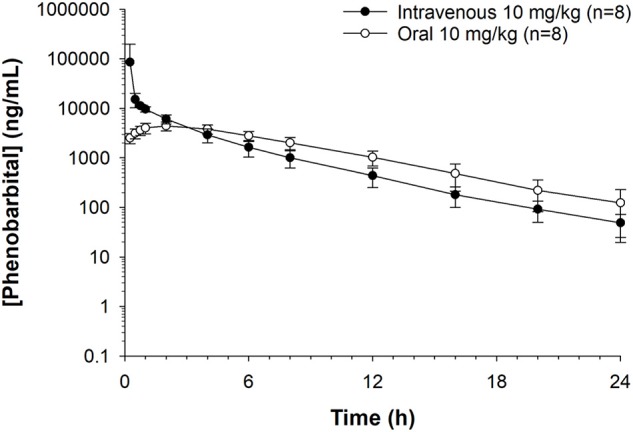
Phenobarbital concentrations plotted against time for oral or intravenous route administration at 10 mg/kg in healthy goats (*n* = 8). Peak plasma phenobarbital concentration after intravenous administration = 34,094 ng/mL. Peak plasma phenobarbital concentration after oral administration = 4,478 ng/mL.

Median (range) bodyweight for goats was 70.8 (56.5–85.0) kg. There were no side effects reported based on clinical examination parameters including appetite, and rectal temperature after the administration of phenobarbital. The goats tolerated the placement of the electrodes and maintenance of the EEG system machine. The individual goat phenobarbital concentrations at each time point for the IV and PO routes of administration are summarized in Tables 1, 2, respectively. The phenobarbital concentrations plotted against time for the oral and intravenous routes are depicted in Figure 2. The pharmacokinetic parameters are summarized in Table 3. Bioavailability of phenobarbital was 24.9%, and half-life was similar between the PO (3.8 h) and IV (4.0 h) routes. The C_max_ and T_max_ for the PO route were 4,478.7 ± 962.4 ng/mL and 1.75 ± 0.46 h, respectively. The AUC_0 → ∞_ for the IV and PO routes were 155,813 ± 218,448 and 38,763 ± 9,832 h^*^ng/mL, respectively.

**Table 1:** Individual and average plasma concentrations following intravenous administration of phenobarbital at 10 mg/kg in 8 goats.

**Time (h)**	**Goat 1**	**Goat 2**	**Goat 3**	**Goat 4**	**Goat 5**	**Goat 6**	**Goat 7**	**Goat 8**	**Mean ± SD**
**Concentration (μg/mL)**									
0	ND	ND	ND	ND	ND	ND	ND	ND	ND
0.25	20.93	142.08	343.64	25.66	27.70	44.13	45.40	34.97	85.56 ± 111.36
0.5	11.67	15.89	26.84	13.87	14.66	14.06	11.64	12.76	15.17 ± 4.93
0.75	10.05	11.07	11.79	11.59	12.46	10.92	11.29	11.19	11.30 ± 0.70
1	7.05	8.99	9.90	10.12	10.70	10.33	9.90	10.25	9.65 ± 1.16
2	3.47	6.23	5.37	6.51	7.15	7.59	6.46	6.26	6.13 ± 1.26
4	0.90	2.96	2.69	3.18	3.98	3.34	2.90	3.43	2.92 ± 0.91
6	0.30	1.66	1.42	1.95	2.26	1.93	1.55	2.05	1.64 ± 0.61
8	0.17	0.86	0.93	1.24	1.34	1.30	1.00	1.23	1.01 ± 0.38
12	0.05	0.37	0.38	0.61	0.51	0.65	0.40	0.55	0.44 ± 0.19
16	0.01	0.13	0.18	0.26	0.22	0.26	0.17	0.19	0.18 ± 0.08
20	0.00	0.06	0.11	0.12	0.10	0.15	0.09	0.10	0.09 ± 0.04
24	0.00	0.03	0.06	0.07	0.05	0.08	0.05	0.05	0.05 ± 0.02

*ND, not detected*.

**Table 2:** Individual and average plasma concentrations following oral administration of phenobarbital at 10 mg/kg in 8 goats.

**Time (h)**	**Goat 1**	**Goat 2**	**Goat 3**	**Goat 4**	**Goat 5**	**Goat 6**	**Goat 7**	**Goat 8**	**Mean ± SD**
**Concentration (μg/mL)**									
0	ND	ND	ND	ND	ND	ND	ND	ND	ND
0.25	1.68	2.23	2.79	2.65	3.64	2.57	2.59	2.03	2.52 ± 0.58
0.5	2.05	3.12	3.77	3.19	4.39	3.17	2.78	2.64	3.14 ± 0.71
0.75	2.36	3.38	4.17	3.91	4.89	3.78	3.43	3.03	3.62 ± 0.76
1	2.57	3.81	4.42	3.73	5.99	4.37	3.71	3.57	4.02 ± 0.98
2	2.54	4.14	4.97	4.68	5.72	4.80	4.30	4.38	4.44 ± 0.91
4	2.00	3.74	3.90	4.48	4.44	4.44	3.87	3.74	3.83 ± 0.80
6	1.36	2.70	2.59	3.31	3.30	3.25	2.96	2.91	2.80 ± 0.64
8	0.82	2.05	1.66	2.37	2.50	2.46	2.20	2.09	2.02 ± 0.56
12	0.32	0.92	0.89	1.39	1.29	1.29	1.15	0.99	1.03 ± 0.34
16	0.10	0.32	0.39	1.03	0.48	0.63	0.50	0.42	0.48 ± 0.27
20	0.03	0.14	0.20	0.52	0.22	0.25	0.24	0.17	0.22 ± 0.14
24	0.01	0.07	0.10	0.37	0.10	0.13	0.12	0.09	0.12 ± 0.10

*ND, not detected*.

**Table 3:** Non-compartmental pharmacokinetic parameters, reported as mean ± standard deviation (SD) in healthy goats administered phenobarbital at 10 mg/kg intravenously or orally in a cross-over design (*n* = 8).

**Parameter**	**Intravenous route (*n* = 8)** **(Mean ± SD)**	**Oral route (*n* = 8)** **(Mean ± SD)**
C_max_ (ng/mL)	–	4,478.7 ± 962.4
T_max_ (h)	–	1.75 ± 0.46
λ*_*z*_* (1/h)	0.177 ± 0.027	0.189 ± 0.037
HL λ*_*z*_* (h)	4.0 ± 0.619	3.80 ± 0.826
Cl (ml/h/kg)	152.5 ± 102.7	–
V_ss_ (L/kg)	354 ± 232	–
AUC_0 → ∞_ (h*ng/mL)	155,813 ± 218,448	38,763 ± 9,832

*Oral bioavailability (F) = 0.249 (24.9%)*.

*C_max_, maximum plasma concentration*.

*T_max_, time to observed maximum concentration*.

*λ_z_, terminal-phase rate constant*.

*HL λ_z_, terminal-phase half-life*.

*Cl, clearance*.

*V_ss_, apparent volume of distribution at steady state*.

*AUC_0 → ∞_, area under the curve from zero to infinity*.

**Figure 3:**
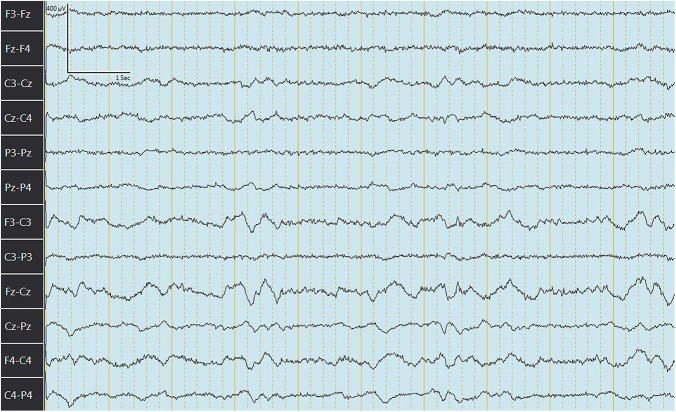
Electroencephalogram depicting slow wave sleep (SWS) in a goat after administration of phenobarbital. Note the slowing of the waves.

**Figure 4:**
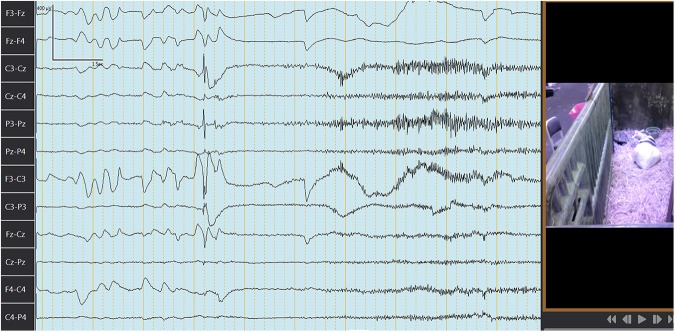
Electroencephalogram depicting rapid eye movement (REM) sleep in a goat after administration of phenobarbital. The first 4 s here: characteristic rapid eye movement and lack of muscle tone followed by awakening and movement (note change in waves frequency and amplitude, movement artifacts, and lack of REM). Channels closest to the eyes are picking up REM (F3-Fz, Fz-F4, F3-C3, F4-C4, and Fz-Cz). Each darker continuous vertical line represents 1 s. Figure on the right represents concurrent video recording demonstrating goat sleeping with head on the ground during REM sleep.

The median (range) behavior scores observed concurrently with EEG recordings in goats before and after IV or PO administration are summarized in Table 4. Figures 3, 4 depict an EEG demonstrating slow wave sleep and rapid eye movement sleep, respectively. Behavior score at 3 h after the administration of phenobarbital was different (*P* = 0.0002) from the score prior to administration (median score = 4 at 3 h after administration vs. median score = 1 prior to administration) for the PO route. Behavior scores at all other time points after administration of phenobarbital were not different (*P* > 0.05 for all comparisons) from the scores prior to administration for the PO route. Behavior scores before administration of phenobarbital and each time point after administration were not different (*P* > 0.05 for all comparisons) for the IV route.

**Table 4:** Median (range) behavior scores observed concurrently with electroencephalogram (EEG) recordings at each time point in healthy goats before and after intravenous or oral administration of phenobarbital (*n* = 8).

**Time**	**Intravenous route**	**Oral route**
BL	1 (1, 1)	1 (1, 1)
PB	1 (1,1)	1 (1–2)
T15	1 (1–4)	1 (1–4)
T30	1.5 (1–4)	2 (1–5)
T45	1.5 (1–3)	1.5 (1–4)
T1	2 (1–5)	1.5 (1–4)
T2	1 (1–4)	2 (1–6)
T3	1 (1–5)	4 (2–6)
T4	1 (1–2)	2 (1–5)
T5	1 (1–4)	3 (1–6)
T6	1 (1–4)	2 (1–5)
T7	1.5 (1–6)	2 (1–5)
T8	1 (1–2)	2 (1–4)
T9	1 (1–5)	2 (1–6)
T10	1 (1–4)	2 (1–5)
T11	1 (1–4)	2.5 (1–5)
T12	1 (1, 1)	1 (1–5)

*Scoring scale:*.

*Score 1 = Awake moving (EEG; high frequency, low amplitude, mixed with gamma frequency, or non-interpretable EEG due to movement or chewing)*.

*Score 2 = Quiet sternal (high frequency, low amplitude with no gamma frequency)*.

*Score 3 = Drowsy (quiet sternal with eyes semi-closed, high frequency, low amplitude with some slowing of the waves)*.

*Score 4 = Transients of sleep (defined as isolated waves distinguished from background activity, and predominant slowing of the waves)*.

*Score 5 = Slow wave sleep (SWS; slow waves, sleep spindles, delta frequency, recumbency)*.

*Score 6 = Rapid eye movement sleep (REM; high frequency, low amplitude with rhythmic rapid eye movements, recumbency with head supported on ground, fence or other pen structures, and lack of muscle tone)*.

*BL, baseline (prior to phenobarbital administration); PB, phenobarbital administration; T15 = 15 min post administration; T15 = 15 minutes post administration, T30 = 30 min, T45 = 45 min, T1 = 1 h, T2 = 2 h, T3 = 3 h, T4 = 4 h, T5 = 5 h, T6 = 6 h, T7 = 7 h, T8 = 8 h, T9 = 9 h, T10 = 10 h, T11 = 11 h, and T12 = 12 h*.

## Discussion

Our study findings indicate that the bioavailability of orally administered phenobarbital was very poor in goats, contrary to our hypothesis. The maximum plasma concentrations achieved with PO dosing of phenobarbital was significantly lower compared to the IV route. Bioavailability of a single, PO dose of phenobarbital in dogs ranged from 86 to 96% when administered at 10 mg/kg (2) and 76 −124% in horses when administered at 5.5 mg/kg (9). Our study results suggest that doses >10 mg/kg should be considered for oral administration in goats to induce higher drug concentrations exposure. Phenobarbital is a lipid soluble, weak acid (11), but the rumen pH is relatively alkaline, ranging from 6.2 to 7.2 (10). The alkaline pH in the rumen potentially enhanced the solubility of phenobarbital but decreased its absorption due to increased ionization (12). Thus, in our study the absorption of phenobarbital was most likely decreased by the alkaline pH in the rumen. Additionally, degradation of phenobarbital by rumen microbes may have occurred thereby reducing the available drug for absorption.

The half-life of phenobarbital in our study was short and consistent between the IV (4 h) and PO (3.8 h) routes. The half-life was substantially lower than 56 and 52 h reported for IV and PO, respectively, in dogs (2) or 11.4 and 19 h for IV and PO, respectively, in horses (3, 9). The short half-life of phenobarbital in our study is also consistent with the high magnitude of clearance. Clearance (152.5 ml/h/kg) of phenobarbital in our study was substantially higher than in humans (3.75–4.2 ml/h/kg) (13, 14), dogs (7–13 ml/h/kg) (15, 16) and horses (27.9 ml/h/kg) (9). The reason for high clearance of phenobarbital in goats compared to other species remains unknown based on our study results, but possible reasons include a higher intrinsic hepatic clearance or greater renal clearance. Drugs that are <80–95% protein bound can penetrate tissues better but are excreted faster (17). Protein binding in serum of phenobarbital in goats is 34–52% consistent with other veterinary species including sheep (24–30%), cattle (4–23%), pigs (10–25%), horses (31%) (18) and dogs (45%) (19). Therefore, the increased magnitude in clearance of phenobarbital in goats compared to horses and dogs cannot be explained by the low plasma protein binding alone. Consequently, intrinsic hepatic clearance might explain the increased clearance and short half-life of phenobarbital in goats. While once daily dosing of phenobarbital in horses at 11 mg/kg (9), and twice daily dosing at 2 mg/kg in dogs (20) has been recommended for managing epilepsy, our study results indicate that once, oral daily dose at 10 mg/kg in goats is likely insufficient for consideration to manage epilepsy due to the high clearance and short half-life. The wide standard deviation in the AUC for both IV and PO routes (Table 3) suggest a significant variability in drug concentrations or total exposure of phenobarbital in goats, which might subsequently affect monitoring response to therapy. The wide variability in phenobarbital disposition in our study is consistent with studies in dogs (20, 21) and horses (3). Further studies are required to assess whether monitoring protocols recommended for dogs including measuring serum phenobarbital concentrations after 2 weeks of administration to assess drug levels are applicable to goats. Therapeutic levels of phenobarbital have not been established in veterinary medicine but are extrapolated from human medicine. However, this extrapolation might not be applicable to veterinary species.

Goats in our study showed behavior score changes concurrently with changes in vigilance based on EEG recordings at 3 h after PO administration of phenobarbital, but not at any other time points for either PO or IV routes. This behavior change time point is at 1 h after the T_max_ and is close to but less than the half-life of phenobarbital and might indicate the time period when the possible maximum drug had been absorbed resulting in the observed behavioral changes. Our results suggest that monitoring behavior changes concurrently with EEG recordings should be considered in goats when phenobarbital is administered orally. Furthermore, this study also showed that the use of the ambulatory EEG is feasible in goats.

Our study has limitations. Despite appropriate flushing with heparinized saline and single use of needles and syringes, using the same catheter for phenobarbital administration and blood collection might potentially result in falsely elevated phenobarbital concentrations for the IV route. We only assessed single dose pharmacokinetics of phenobarbital because our focus was to obtain baseline information in goats. In clinical practice, goats with epilepsy are administered multiple doses of phenobarbital. Thus, our study results might have limited external validity for goats administered multiple doses of phenobarbital. Our study only focused on the qualitative analysis of the raw EEG data concurrently with visual inspection of continuous video recording and designed a scoring system that could be easily applied to clinical practice. Further studies assessing quantitative EEG parameters may be useful. Quantitative analysis was not performed in this study due to the presence of multiple artifacts from movement, chewing or rumination that prevented obtaining an interpretable EEG recording. The goats used in our study were clinically healthy and clinical trials assessing administration of phenobarbital in goats with epilepsy are warranted. Administration of phenobarbital in goats results in residues in meat and milk. The anecdotal recommended withdrawal time for meat and milk in our study was 180 days (22). Thus, owners should be made aware of the residue withdrawal times for phenobarbital when administered in food producing goats. Further studies assessing pharmacokinetics of different dosing >10 mg/kg, multiple dosing, assessing quantitative EEG parameters, pharmacodynamics studies, and determination of therapeutic range in goats with epilepsy are required.

## Conclusions

Oral bioavailability of phenobarbital in goats is poor (24.9%). The half-life (3.8–4 h) of phenobarbital in goats is very short due to its higher clearance. There is a wide individual variability in disposition of phenobarbital in goats. Concurrent qualitative assessment of behavior and EEG recordings using a scoring system created in our study should be considered for monitoring goats administered phenobarbital orally.

## Data Availability Statement

The datasets generated for this study are available on request to the corresponding author.

## Ethics Statement

The animal study was reviewed and approved by the University of California Davis Animal Use and Care Committee (UCD protocol #21258).

## Author Contributions

LY, MC, and MA conceived the work, designed the study, performed data analysis, and wrote the manuscript. LY and MC prepared for one of the grants. LY, MC, MK, and CC conducted the work and collected the research data. HK performed samples analysis. All authors reviewed the data analysis and approved the final manuscript.

### Conflict of Interest

The authors declare that the research was conducted in the absence of any commercial or financial relationships that could be construed as a potential conflict of interest.

## Abbreviations

δ: Delta frequency band (>0 to <4 Hz)
θ: Theta frequency band (4 to <8 Hz)
α: Alpha frequency band (8 to <13 Hz)
β: Beta frequency band (13 to <30 Hz)
γ: Gamma frequency band (>30 Hz)
BL: Baseline
EEG: Electroencephalogram
IV: Intravenous
PB: Phenobarbital
PO: Oral
REM: Rapid eye movement
SWS: Slow wave sleep.
